# The Morphologically Controlled Synthesis and Application of Mesoporous Alumina Spheres

**DOI:** 10.3390/molecules28155622

**Published:** 2023-07-25

**Authors:** Yadian Xie, Lanxing Gao, Miaoxuan Xue, Yanqing Hou, Bo Yang, Lingyun Zhou, Xin Tong

**Affiliations:** 1 Guizhou Provincial Key Laboratory in Higher Education Institutions of Low-Dimensional Materials and Environmental and Ecological Governance, Key Laboratory of Low-Dimensional Materials and Big Data, College of Chemical Engineering, Guizhou Minzu University, Guiyang 550025, China; glxprettylife@163.com (L.G.); xuemx0418@163.com (M.X.); hhouyanqing@163.com (Y.H.); gznuyangbo@163.com (B.Y.); 2 Faculty of Metallurgy and Energy Engineering, Kunming University of Science and Technology, Kunming 650093, China; 3 School of Chemistry and Material Science, Guizhou Normal University, Guiyang 550001, China

**Keywords:** mesoporous alumina, chitin, P123, hydrothermal method

## Abstract

The control of alumina morphology is crucial yet challenging for its various applications. Unfortunately, traditional methods for preparing alumina particles suffer from several limitations such as irregular morphology, poor dispersibility, and restricted application areas. In this study, we develop a novel method for preparing spherical mesoporous alumina using chitin and Pluronic P123 as mixed templates. The effects of reaction temperature, time, and the addition of mixed templates on the phase structure, micromorphology, and optical absorption properties of the samples were investigated. The experimental results indicate that lower temperature and shorter reaction time facilitated the formation of spherical mesoporous alumina with excellent CO_2_ adsorption capacity. The periodic density functional theory (DFT) calculations demonstrate that both the (110) and (100) surfaces of γ-Al_2_O_3_ can strongly adsorb CO_2_. The difference in the amount of CO_2_ adsorbed by Al_2_O_3_ is mainly due to the different surface areas, which give different numbers of exposed active sites. This approach introduces a novel strategy for utilizing biological compounds to synthesize spherical alumina and greatly enhances mesoporous alumina’s application efficiency in adsorption fields. Moreover, this study explored the electrochemical performance of the synthesized product using cyclic voltammetry, and improved loading of electrocatalysts and enhanced electrocatalytic activity were discovered.

## 1. Introduction

In recent years, alumina has gained significant attention in high-value applications such as adsorbent [1,2,3], ceramic [4,5], catalyst and catalyst carrier [6,7,8,9] applications, etc. The application of alumina is not only dependent on particle size but also on particle shape. Different shapes of alumina, such as rod-like [10], fibrous [11], plate-like [12], and spherical [13] shapes, have been wildly used. Seyed [14] obtained massive γ-aluminum with particle sizes between 0.5 μm and 0.9 μm, but the morphology was irregular. Dabbagh et al. and Feng et al. [15,16] prepared rod-like, fibrous, and spherical alumina, but the process was complex and hard to control, resulting in poor dispersibility. Lv et al. [17] synthesized spherical alumina with a highly spherical shape and uniform particle size by using the oil–ammonia drop method, but the particle size was large, which limited its application area. Using aluminum isopropoxide as a precursor and Pluronic P123 (P123) as a template, Wu et al. [18] synthesized organized mesoporous alumina with a hierarchical structure. However, these methods suffer from limitations such as irregular morphology, poor dispersibility, and limited application areas.

Mesoporous materials are highly valued due to their higher specific surface area, organized pore structure, narrow pore size distribution, and continuous pore size, which make them important for adsorption and separation, as well as catalytic reactions [19,20,21,22,23]. Among various shapes of alumina, spherical alumina has high fluidity, making it less prone to gathering and producing channeling during catalytic processes, which significantly enhances catalyst activity. Therefore, the preparation of mesoporous alumina can greatly improve the application efficiency of mesoporous alumina spheres in adsorption, separation, and catalysis [24].

Chitin ((C_8_H_13_O_5_N)_n_) is a naturally occurring biopolymer that is highly organized and abundant in the exoskeletons of crustaceans and insects. The primary chitin fibrils’ structural arrangement varies among arthropod species, with some having helical structures called the Bouligand structure, which enhances photonic and mechanical properties. This structure resembles cholesteric lyotropic liquid crystals. The hierarchical structure of chitin fibrils makes them an excellent natural template for developing new materials. The use of biomass as a porous material can achieve biodegradability, achieve biocompatibility, and achieve green and sustainable development [25,26,27,28,29]. Pluronic P123 (P123) is a soft template with a symmetric triblock copolymer comprising poly (ethylene oxide) (PEO) alternating with poly (propylene oxide) (PPO), PEO-PPO-PEO. Its phases vary depending on the concentration and combination of solvents [30,31,32,33] and it is often used as a crystal structure modifier in the preparation of mesoporous materials [34].

In this study, mesoporous alumina spheres were successfully prepared using alumina hydrate (AlOOH) as a precursor, (NH_2_)_2_CO as a precipitant, and chitin powder and P123. The preparation process, which involved evaporation-induced self-assembly (EISA), was green, low-cost, and pollution-free. This method significantly improved the catalyst loading firmness and service life of mesoporous alumina spheres. The effects of synthesis temperature, time, and the addition of mixed templates on the structure and morphology of the products were investigated. The CO_2_ adsorption performance of spherical mesoporous alumina and the electrochemical performance of supported SnO_2_ were also evaluated.

## 2. Results and Discussion

**Figure 1:**
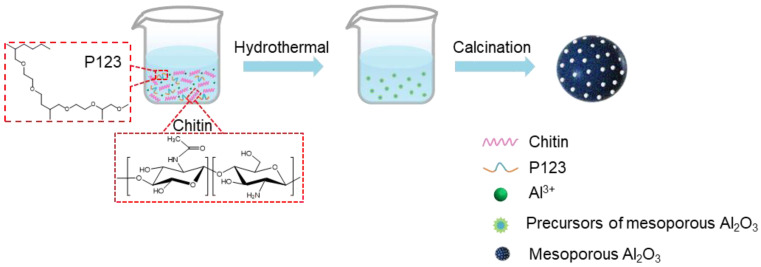
Schematic diagram of the preparation of spherical mesoporous alumina materials.

**Synthesis of mesoporous alumina spheres.** The synthesis of spherical macroporous alumina materials was achieved through the hydrothermal method, using hydrated alumina (AlOOH) as the precursor, along with chitin and P123 as the templates, and urea as a precipitant, while adapting the evaporation-induced self-assembly (EISA) method, as depicted in Figure 1. By systematically varying the reaction conditions, the impact of reaction temperature and time, as well as the chitin/P123 weight ratio, on the morphology and properties of the resulting spherical mesoporous alumina materials was investigated. The detailed process parameters are shown in the following table. During the calcining process at 700 °C, the organic components, including chitin and P123, underwent decomposition and evaporation, creating voids within the alumina matrix. This decomposition and evaporation of organic materials resulted in the formation of well-defined mesopores within the macroporous alumina microspheres.

**Figure 2:**
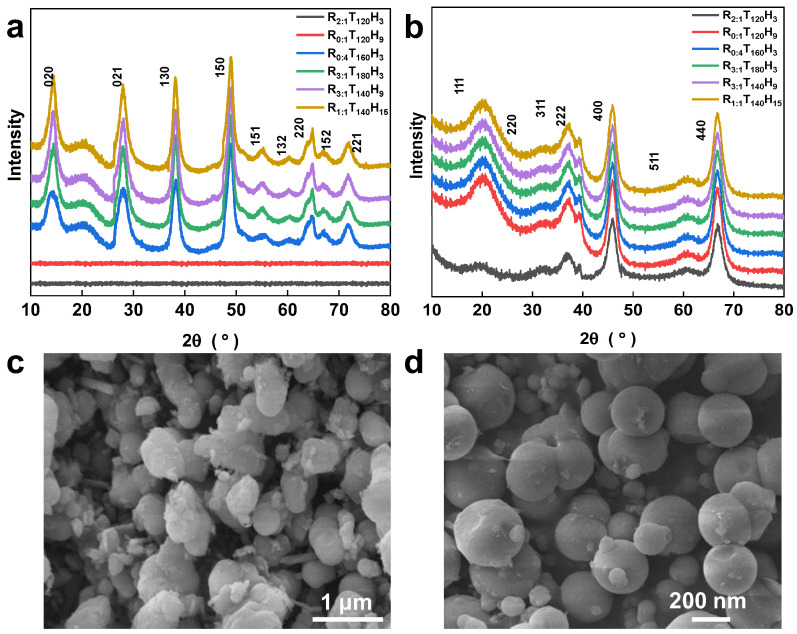
The X-ray diffraction (XRD) patterns of (**a**) the precursor and (**b**) mesoporous alumina XRD image under different synthesis conditions. The SEM images of mesoporous alumina under different synthesis conditions: (**c**) 120 °C, (**d**) 140 °C.

**The effect of synthesis temperature and time on the morphology of spherical mesoporous alumina.** Figure 2a displays the X-ray diffraction (XRD) map of the precursor synthesized under various temperatures and durations. The precursors of R_2:1_T_120_H_3_ and R_0:1_T_120_H_9_ are primarily amorphous products. However, as the synthesis temperature increases to 160 °C and 180 °C, the precursors of R_0:4_T_160_H_3_ and R_3:1_T_180_H_3_ start to crystallize and become AlOOH (JCPDS 01-072-0359). This finding suggests that the synthesis temperature has a significant impact on the crystallization of the precursor. At lower hydrothermal temperatures, the system energy is insufficient to facilitate the formation and transformation of crystals, leading to the amorphous state of the samples. In contrast, when the hydrothermal temperature increases, individual diffraction peaks emerge, and their intensity and width grow, indicating the increased crystallinity of the particles. The precursors of R_3:1_T_140_H_9_ and R_1:1_T_140_H_15_ are synthesized at 140 °C for 9 h and 15 h, respectively, providing sufficient energy for crystallization. Nevertheless, the diffraction peak intensity and width of the precursors of R_3:1_T_140_H_9_ and R_1:1_T_140_H_15_ are smaller compared with those of the precursors of R_0:4_T_160_H_3_ and R_3:1_T_180_H_3_ synthesized at higher temperatures. This difference implies that the crystallinity of particles in the precursors of R_3:1_T_140_H_9_ and R_1:1_T_140_H_15_ is weaker than that in the precursors of R_0:4_T_160_H_3_ and R_3:1_T_180_H_3_. By controlling the growth rate of the crystal faces, the crystal orientation can be influenced, enabling control over the morphology and crystal structure of products.

γ-Al_2_O_3_ belongs to the transitional form of alumina, and the random distribution of Al atoms results in a certain broadening of the XRD diffraction peak of γ-Al_2_O_3_. Figure 2b shows the mesoporous alumina XRD image after calcination at 600 °C. The amorphous precursor of R_2:1_T_120_H_3_ and R_0:1_T_120_H_9_ prepared at a lower synthesis temperature transforms into γ-Al_2_O_3_ under calcination at 700 °C; samples R_0:4_T_160_H_3_, R_3:1_T_180_H_3_, R_3:1_T_140_H_9_, and R_1:1_T_140_H_15_ are γ-Al_2_O_3_ (JCDPS 10-0425). With the increase in hydrothermal temperature, the position of each diffraction peak remains the same. However, the intensity and width of the diffraction peak grow larger, indicating an increase in the crystallinity of particles.

Figure 2c,d illustrates the SEM images of the mesoporous alumina samples prepared under different synthesis conditions. The images reveal that the temperature significantly influences the sample morphology, and 140 °C promotes the formation of spherical particles. When synthesized at 120 °C, the sample particles are massive and spherical (Figure 2c), while at 140 °C, the particles are regularly spherical with a smooth surface and particle sizes ranging between 50 nm and 200 nm (Figure 2d). As the synthesis temperature increases, more particles begin to crystallize and form crystalline solids. While these solids can completely transform into γ-Al_2_O_3_ at 700 °C, the amorphous precursor requires more energy to undergo phase transformation at this temperature. Thus, crystallization is relatively slow, which favors the preparation of spherical mesoporous alumina (Figure 2a,b). However, at higher synthesis temperatures, the chitin powder used as a template tends to carbonize and lose some of its templating function. As a result, the sample transforms into irregular and flocculent particles that agglomerate (Figure S1a–d). Moreover, longer synthesis times are also not conducive to the development of spherical alumina particles (Figure S1c,d). Such conditions promote the development of flocculent, strip, and irregular particles, which hinder the normal growth of spherical particles.

**Figure 3:**
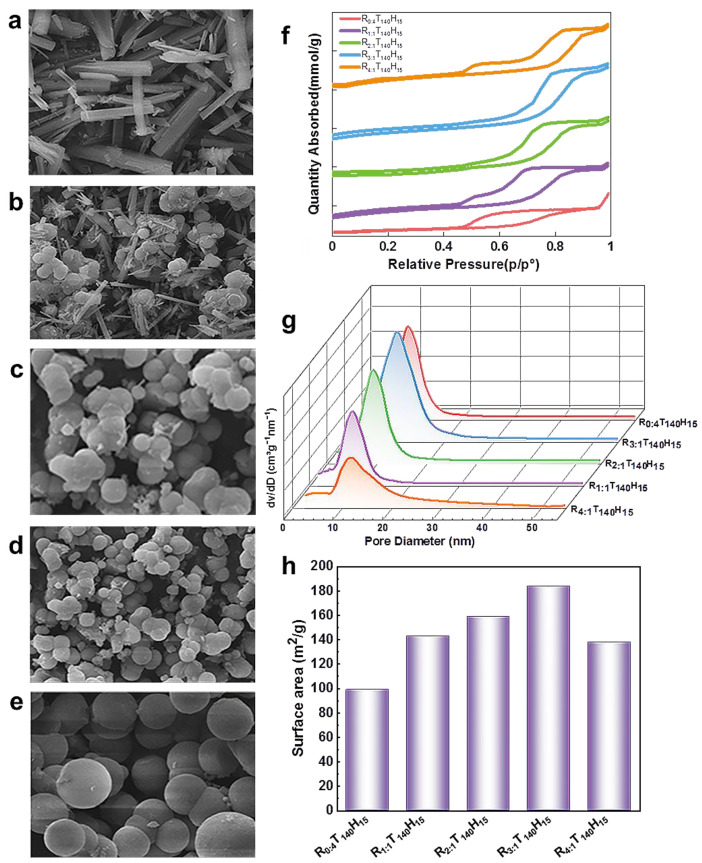
SEM images of (**a**) R_0:4_T_140_H_15_, (**b**) R_1:1_T_140_H_15_, (**c**) R_2:1_T_140_H_15_, (**d**) R_3:1_T_140_H_15_, and (**e**) R_4:1_T_140_H_15_, (**f**) nitrogen sorption isotherms curves, (**g**) corresponding pore size distributions curves and (**h**) surface area histogram of as-prepared samples.

**The effect of chitin/P123 weight ratio on spherical morphology.** During the synthesis process, the amount of additive used has a significant impact on the morphology and structure of the final product [35,36,37,38]. Figure 3 displays the test results of spherical mesoporous alumina prepared using different chitin/P123 weight ratios (chitin quality:P123 quality).

When the chitin/P123 weight ratio is 0 (R = 0:4), the predominant types of pores observed in the samples are inkbottle-type pores with a large opening and small diameter, as well as uneven crack-like pores, as shown in Figure 3a. The nitrogen adsorption isotherm (see Figure 3f) exhibits a type IV curve with a hysteresis loop between H2 and H3 in the middle to high voltage range of 0.5 p/p^0^ to 0.9 p/p^0^. The adsorption amount in this range is limited to only 0.4 to 1 mmol/g. This is primarily due to the irregular stacking of the strip-shaped samples, which results in a low number of mesoporous structures being distributed. As shown in Figure 3g, the pore sizes are mainly distributed in the range of approximately 7 nm.

As the weight ratio increases to 3, 4 (R = 3:1, 4:1), the particle size of the sample becomes larger and the spherical shape becomes more regular (see Figure 3d,e). The nitrogen adsorption isotherms show that in the middle–high pressure area, the nitrogen adsorption capacity has further increased to about 7–8 mmol/g, as shown in Figure 3f,g. The pore size has further increased to about 9.4 nm and the pore volume has increased to 0.7 cm^3^/g. The type IV curve of nitrogen adsorption isotherms shows a hysteresis loop between types H1 and H2. The formation of inkbottle pores becomes more prominent, and the mesoporous structure becomes more developed. However, with the further increase in the weight ratio, the nitrogen adsorption capacity gradually decreases, indicating that excessive chitin may hinder the formation of the mesoporous structure. Therefore, the optimal weight ratio of chitin/P123 is 3, 4 (R= 3:1, 4:1).

As shown in Figure 3d, at a weight ratio of 3 (R = 3:1), the sample formed regular, uniform, and monodisperse microspheres and the predominant pore structure is a regular cylindrical shape with uniform size and shape. The nitrogen adsorption isotherms for this sample show a steep type IV curve and an H1 hysteresis loop (see Figure 3f), indicating that the sample has a uniform mesoporous structure and pore sizes are mainly distributed between 7 nm and 9 nm. Notably, the pore size has increased by 8.6 nm compared with the previous weight ratio, and the pore volume has also increased by 0.3 cm^3^/g.

In summary, the chitin/P123 weight ratio has a significant effect on the morphology and structure of the mesoporous alumina sample. When the weight ratio is 0 (R = 0:4), the sample has irregular strips with few mesoporous structures. As the weight ratio increases to 3 (R = 3:1), the sample forms regular and uniform microspheres with a uniform mesoporous structure, which leads to increases in pore size, pore volume, and BET surface area. However, when the weight ratio is further increased to 4 (R = 4:1), the sample exhibits adhesion and aggregation, and the pore size, pore volume, and BET surface area decrease. This is due to the increase in viscosity of the solution, which affects the template space steric effect and weakens homogeneous nucleation.

Using this method, this experiment involved successfully preparing spherical mesoporous alumina with uniform pore size, good dispersion, and identical morphology, with a chitin/P123 weight ratio of 3 (R = 3:1). The amorphous precursor was prepared at a lower temperature of 140 °C and for a shorter time of 3 h. Upon calcination at 600 °C, inorganic Al^3+^ interacted slowly with and connected to the organic micelle interface via electrostatic interaction. This process allowed the aluminum ions to cover the entire particle surface and achieve consistent growth rates for all surfaces, ultimately resulting in the formation of spherical particles via homogeneous nucleation. The particle surface also formed a uniform mesoporous layer, resulting in the complete transformation of the precursor into spherical mesoporous Al_2_O_3_, as shown in Figure S2.

Figure S2 displays TEM images of spherical mesoporous alumina materials. The images clearly show that the alumina has a uniform and regular spherical form, as well as good dispersion. A 10 nm mesoporous layer uniformly covers the surface of the alumina particles, and the mesopore size is consistent throughout. The nitrogen sorption isotherms and corresponding pore size distributions of the spherical mesoporous alumina materials are also presented in Figure S2.

**Figure 4:**
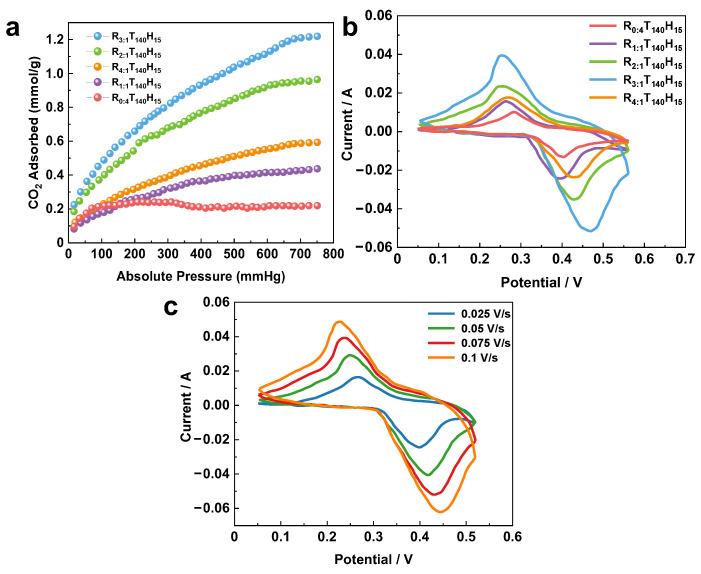
(**a**) CO_2_-adsorption isotherms of mesoporous alumina spheres synthesized with different templating agent ratios. Cyclic voltammograms of (**b**) different templating agent ratios and (**c**) different scan rates.

**CO_2_ adsorption on spherical mesoporous alumina.** Figure 4a displays the CO_2_ gas adsorption isotherm curves of spherical mesoporous alumina, which was synthesized with varying mass ratios of chitin to P123 at a temperature of 273 K [39,40,41,42]. With its large specific surface area and pore volume, spherical mesoporous alumina exhibits a high potential for gas adsorption. In this study, we investigated the adsorption capacity of spherical mesoporous alumina, synthesized with different ratios of template agents (chitin/P123), for carbon dioxide at 273 K.

The adsorption capacity of CO_2_ is highest (1.21 mmol/g) when the ratio of template agents is 3, resulting in the preparation of spherical mesoporous alumina with excellent dispersion and uniform pore size. Notably, the different mass ratios of template agents used in the synthesis produced alumina with distinct shapes, which significantly impacted their adsorption capacities.

**Electrochemical performance of porous alumina-supported SnO_2_.** The electrochemical performance of porous alumina-supported SnO_2_ was studied using spherical mesoporous alumina-supported SnO_2_ particle electrodes [43,44]. The electrodes were prepared using a dip-calcination method, with a loading amount of 4%. Cyclic voltammetry tests were carried out using a three-electrode system with 5 mol/L KOH solution as the electrolyte, a mercury/mercury oxide electrode as the reference electrode, and a platinum electrode as the auxiliary electrode. The prepared electrode sheet was used as the working electrode.

The CV curves of spherical mesoporous alumina-supported SnO_2_ prepared with different ratios of template agents (chitin/P123) at a scan rate of 0.075 V/s are shown in Figure 4b. The redox peak in Figure 4b indicates the pseudocapacitive properties of the material. It was observed that when the ratio of the template agent increases, the area of the cyclic voltammetry curve increases, and the specific capacitance increases significantly, indicating that the spherical mesoporous alumina has a better energy storage performance. However, when the ratio of the template agent is too large (R = 4:1), the viscosity of the solution increases significantly. This increase in viscosity can affect the steric effect of the template space and weaken the homogeneous nucleation, which can lead to poor dispersion of particles. Poor particle dispersion can result in a poor carrier effect, which can negatively impact the electrochemical performance of the material.

The CV curves at different scan rates when the ratio of the template agent (chitin/P123) is 3 and the loading of SnO_2_ is 4% are shown in Figure 4c. The CV curves were observed to be different with different scan rates, and with the increase in the test scan rate, the area of the closed graph also increased. This can be attributed to the high surface area and porous structure of the alumina support, which provides a large number of active sites for SnO_2_ deposition and enhances the catalytic activity of SnO_2_. Mesoporous alumina spheres can be an excellent electrocatalyst support due to their special mesoporous structure.

**Periodic density functional theory (DFT).** To gain deep insight on CO_2_ adsorption, we performed periodic density functional theory (DFT) calculations (see Supplemental Information for computational details) [45,46,47,48,49]. The (110) and (100) surfaces of γ-Al_2_O_3_ were selected, as these two surfaces are predominant in γ-Al_2_O_3_, as proved with XRD in Figure 2b. The optimized structures of Al_2_O_3_(110) and Al_2_O_3_(100) surfaces are shown in Figure S3 [50,51,52,53,54,55,56,57]. The bare Al_2_O_3_(110) surface is terminated with two-coordinated O_(II)_, three-coordinated O_(III)_, and four-coordinated Al_(IV)_, while the Al_2_O_3_(100) surface is exposed with three-coordinated O_(III)_ and five-coordinated Al_(V)_. Our results indicate that the surface energy of Al_2_O_3_(110) is lower than that of Al_2_O_3_(100) (1.41 J·m^−2^ vs. 2.45 J·m^−2^), indicating that the Al_2_O_3_(110) surface is more stable [58,59,60].

**Figure 5:**
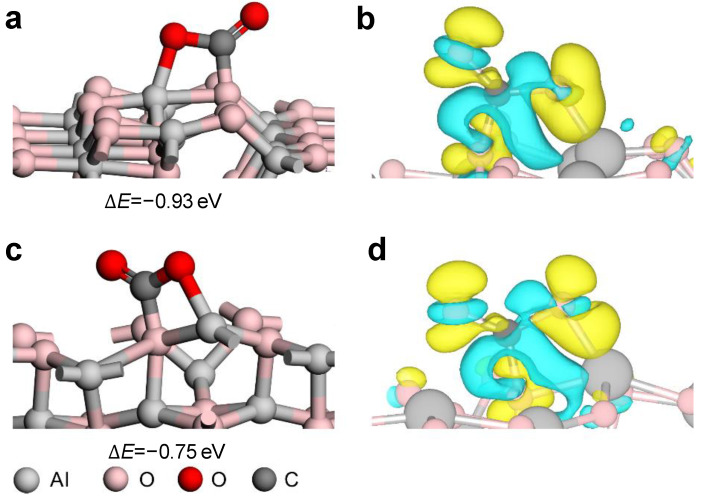
The adsorption energies and optimized structures of CO_2_ on (**a**) Al_2_O_3_(110) and (**b**) Al_2_O_3_(100) surfaces. (**c**,**d**) are the charge differences in CO_2_ adsorption on Al_2_O_3_(110) and Al_2_O_3_(100) surfaces, respectively. The values of the isosurfaces are ±0.003 e/Å^3^. Yellow and light cyan isosurfaces indicate the accumulation and depletion of charge density.

The adsorption energies and optimized structures of CO_2_ on Al_2_O_3_(110) and Al_2_O_3_(100) surfaces are shown in Figure 5 and Figure S4 [61]. The DFT calculated results show that the formation of carbonate species is the most stable adsorption model, where CO_2_ binds in an ambidentate configuration across the O-Al bridge sites with adsorption energies of −0.93 eV and −0.75 eV on Al_2_O_3_(110) and Al_2_O_3_(100), respectively. To obtain insight into the nature of molecular adsorption behavior, the differences in charge density (Δρ) of the most stable adsorption CO_2_ on Al_2_O_3_ surfaces were calculated. The results are clearly plotted in Figure 5. It is evident that charges accumulate and deplete around the O and C of CO_2_ on both surfaces, respectively. The net result is the transfer of electrons from Al_2_O_3_ surfaces to the adsorbed CO_2_. The Bader charge analyses proved that the electronic charges transferred from the Al_2_O_3_(110) and Al_2_O_3_(100) surfaces to CO_2_ are 0.28 a.u. and 0.29 a.u., respectively. These two surfaces achieve the purpose of CO_2_ activation by transferring their electrons to the antibonding molecular orbital of CO_2_. The former is more stable, mainly because there are more electrons near the Fermi level on the atoms of the surface active site of Al_(IV)_-O_(II)_ on Al_2_O_3_(110) than that of Al_(V)_-O_(III)_ on Al_2_O_3_(100) (see projected density of states in Figure S5). The above results demonstrate that both surfaces can strongly adsorb CO_2_, which explains the experimental results. The difference in the amount of CO_2_ adsorbed by Al_2_O_3_ synthesized under different conditions is mainly due to the different surface areas, which give different numbers of exposed active sites. The higher the crystallinity, the higher the surface content of Al_2_O_3_(110) [45].

## 3. Materials and Methods 

### 3.1. Synthesis of Mesoporous Alumina Spheres

The typical synthesis process involves the following steps: Firstly, 6.6 g of hydrated alumina is added to ultrapure water with a resistivity of 18 MΩ·cm, and dissolved in a volumetric flask to prepare a 0.6 mol/L hydrated alumina solution. Subsequently, 3 g of chitin and 1 g of P123 are dissolved in 40 mL of isopropanol, followed by the addition of 2.4 g of urea, and stirred until fully dissolved. Next, 30 mL of the prepared hydrated alumina solution is added and vigorously stirred at room temperature. After 30 min, the solution is transferred into an autoclave with a PTFE liner, and reacted at 140 °C for 3 h. Following this, the product is rapidly cooled to room temperature using a water bath. The synthesized samples are purified via repeated redispersion in ultrapure water followed by filtration to remove any unreacted reagents. The samples are washed with 500 mL of ultrapure water and 200 mL of absolute ethanol, respectively, 3–5 times. The obtained material is then dried under vacuum at 80 °C, and finally calcined at 700 °C for 2 h. The resulting product is a white spherical mesoporous alumina powder. 

The hydrothermal reaction temperatures are 120 °C, 140 °C, 160 °C, and 180 °C. The hydrothermal reaction times are 3 h, 9 h, and 15 h. The chitin/P123 weight ratio is maintained in the range of 0–4. Table 1 shows the process parameters for preparing alumina and spherical mesoporous alumina, respectively. The resultant samples were denoted as R_m_T_n_H_s_ (m = chitin/P123 weight ratio, n (reaction temperature) = 120, 140, 160, or 180 °C, respectively; s (reaction time) = 3, 9, or 15 h, respectively). 

### 3.2. Physical Characterization

The physical properties of the synthesized spherical mesoporous alumina materials were determined using various techniques. The X-ray diffraction (XRD) patterns were obtained using a PANalytical X’Pert PRO diffractometer with Cu Kα radiation (λ = 1.54056 Å) over the 2θ range of 10–80°. The N_2_ adsorption–desorption isotherms were measured at −196 °C using a Micromeritics Tristars 3000 analyzer. The CO_2_ adsorption isotherms of the synthesized spherical mesoporous alumina materials were measured using a Micromeritics Tristars 3000 analyzer instrument at 25 °C. Prior to the measurements, the samples were degassed at 180 °C under vacuum for 6 h to remove any moisture and adsorbed gases. The CO_2_-adsorption isotherms were obtained at various pressures ranging from 0 to 1 bar. The CO_2_ uptake capacity of the materials was calculated from the amount of gas adsorbed at equilibrium and the mass of the sample. The specific surface area (SSA) and pore volume were calculated using the Brunauer–Emmett–Teller (BET) method and Barrett–Joyner–Halenda (BJH) analysis, respectively. The scanning electron microscopy (SEM) images were obtained using a KYKY-2800B microscope. Transmission electron microscopy (TEM) measurements were carried out on Tecnai G2 F20 operated at 200 kV.

### 3.3. Electrochemistry Characterization

The electrochemical performance of the synthesized spherical mesoporous alumina materials was evaluated using a three-electrode system with a platinum wire as the counter electrode, a mercury/mercury oxide electrode as the reference electrode, and the synthesized material as the working electrode. The electrochemical tests were carried out using a CHI660D electrochemical workstation in a 5 mol/L KOH electrolyte solution. Cyclic voltammetry (CV) was carried out in the potential range of 0 to 0.7 V vs. Hg/HgO at a scan rate of 0.025–0.1 V/s to investigate the electrochemical stability of the synthesized materials. 

## 4. Conclusions

In summary, using P123 as a soft template, mesoporous alumina spheres with high specific surface area, larger pore size, and larger pore volume were synthesized. The synthesis conditions, such as temperature, time, and the weight ratio of additives, have a significant impact on the morphology and properties of the material. By optimizing these conditions, monodisperse spherical shape and uniform mesoporous structure can be elicited, leading to better CO_2_ adsorption capacity and improved electrocatalytic effect. This method provides a new way to control pore size and structure, and the material has potential applications in various fields. The periodic density functional theory (DFT) calculation results demonstrate that both the (110) and (100) surfaces of γ-Al_2_O_3_ can strongly adsorb CO_2_, which explains the experimental results. The difference in the amount of CO_2_ adsorbed by Al_2_O_3_ synthesized under different conditions is mainly due to the different surface areas, which give different numbers of exposed active sites. The higher the crystallinity, the higher the surface content of Al_2_O_3_(110). This research contributes to the development of mesoporous materials and enriches the diversity in solution phase synthetic chemistry.

## Figures and Tables

**Table 1:** Detailed process parameters for preparing spherical mesoporous alumina.

Sample	Weight Ratio (Chitin/P123)	Reaction Temperature (°C)	Reaction Time (h)
R_2:1_T_120_H_3_	2:1	120	3
R_0:1_T_120_H_9_	0:1	120	9
R_4:1_T_120_H_15_	4:1	120	15
R_1:2_T_140_H_3_	1:2	140	3
R_3:1_T_140_H_9_	3:1	140	9
R_1:1_T_140_H_15_	1:1	140	15
R_0:4_T_160_H_3_	0:4	160	3
R_2:3_T_160_H_9_	2:3	160	9
R_3:1_T_160_H_15_	3:1	160	15
R_3:1_T_180_H_3_	3:1	180	3
R_1:2_T_180_H_9_	1:2	180	9
R_0:4_T_180_H_15_	0:4	180	15

## Data Availability

Data sharing not applicable. No new data were created or analyzed in this study.

## Supplementary Materials

The following supporting information can be downloaded at: https://www.mdpi.com/article/10.3390/molecules28155622/s1, Table S1: Process parameters of alumina prepared by different reaction temperature and reaction time; Table S2: Process parameters of spherical alumina prepared by different addition of Chitin powder; Table S3: Textural properties of mesoporous alumina with different Chitin:P123 weight ratio; Figure S1: Mesoporous alumina samples SEM image prepared under different synthesis conditions: a: Al_2_O_3_-160-3; b: Al_2_O_3_-180-3; c: Al_2_O_3_-140-6; d: Al_2_O_3_-140-9; Figure S2: TEM images of spherical mesoporous alumina materials; Figure S3: The top view and side view of the computational models. (a) Al_2_O_3_(110) and (b) Al_2_O_3_(100) surfaces; Figure S4: The adsorption energies and optimized structures of CO2 on (a)-(b) Al_2_O_3_(110) and (c) Al_2_O_3_(100) surfaces; Figure S5: Projected density of states (PDOS) of the active sites of Al(IV)-O(II) or Al(V)-O(III) on Al_2_O_3_(110) and Al_2_O_3_(100), respectively. Reference citations of [45,46,47,48,49,50,51,52,53,54,55,56,57,58,59,60,61].
