# Supplementary material for: The Morphologically Controlled Synthesis and Application of Mesoporous Alumina Spheres
Source: Molecules. 2023 Jul 25;28(15):5622. doi: 10.3390/molecules28155622 (PMC10420170; doi:10.3390/molecules28155622)
Supplement: Supplementary file 1 [file molecules-28-05622-s001.zip › molecules-2515322-supplementary.pdf]

Supporting Information

Double Templating Synthesis of Nanoscale - Spherical Mesoporous Alumina

Instrumental Characterization

X ray diffraction (XRD． PANalytical X’Pert PRO． Almelo，Holland) and scanning electron microscope (SEM，KYKY-2800B) were used to study the crystallinity and morphology of prepared powders. Transmission electron microscopy (TEM) measurements were carried out on Tecnai G2 F20 operated at 200 kV. All samples were first dispersed in ethanol and then collected by using copper grid covered with carbon films for measurements. Nitrogen sorption isotherms were measured at 77 K on a Micromeritics Tristars 3000 analyzer. Before measurements, the samples were degassed in a vacuum at 180oC for at least 6 h. The Brumauer–Emmett–Teller (BET) method was utilized to calculate the specific surface areas (SBET), using adsorption data in a relative pressure range from 0.04 to 0.2. The pore volume and pore size distributions were derived from the adsorption branches of isotherms by using the Barrett–Joyner–Halenda (BJH) model.

Table S1. Process parameters of alumina
prepared by different reaction temperature and reaction time

Sample number	Urea (g)	Chitin /P123 Weight ratio	Temperature (°C)	Time (h)
AT1	2.4	3	120	3
BT2	2.4	3	140	3
CT3	2.4	3	160	3
DT4	2.4	3	180	3
ET5	2.4	3	140	9
FT6	2.4	3	140	15

Table S2. Process parameters of spherical alumina prepared by different addition of Chitin powder

Sample number	Urea (g)	Chitin/P123 Weight ratio	Temperature (℃)	Time (h)
G	2.4	0	140	3
H	2.4	1	140	3
I	2.4	2	140	3
J	2.4	3	140	3
K	2.4	4	140	3

Table S3. Textural properties of mesoporous alumina with different Chitin:P123 weight ratio.

Sample	Weight ratio (Chitin:P123)	Pore size (nm)	Pore volume (cm^3/g)	BET surface area (m^2/g)
$R_{0:4}T_{140}H_{15}$	0:4	7	0.04	99
$R_{1:1}T_{140}H_{15}$	1:1	7.6	0.17	143
$R_{2:1}T_{140}H_{15}$	2:1	7.6	0.3	159
$R_{3:1}T_{140}H_{15}$	3:1	8.6	0.3	184
$R_{4:1}T_{140}H_{15}$	4:1	5.1	0.23	138

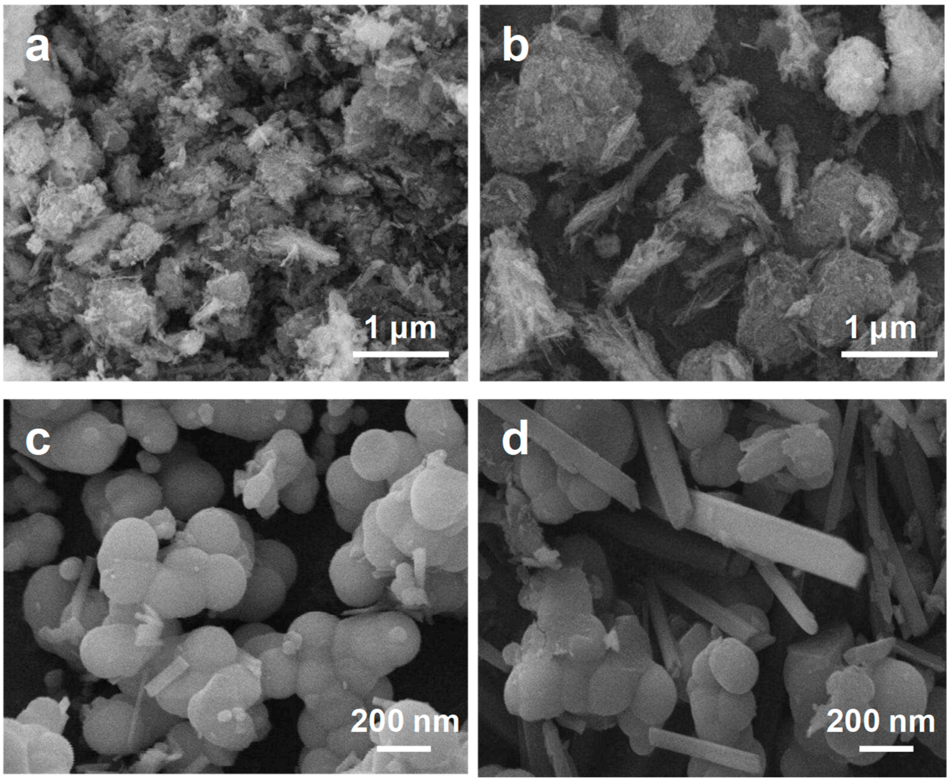

Figure S1. Mesoporous alumina samples SEM image prepared under different synthesis conditions: a: Al_2O_3-160-3; b: Al_2O_3-180-3; c: Al_2O_3-140-6; d: Al_2O_3-140-9.

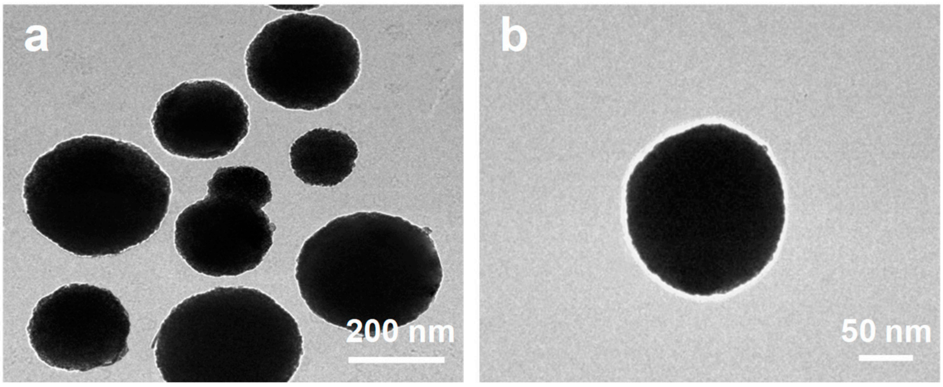

Figure S2. TEM images of spherical mesoporous alumina materials.

Computational details

Computational methods

We used density functional theory (DFT) executed in the Vienna ab initio simulation package (VASP6.3.3) for all the calculations.[46–49] The valence electrons were described by a plane wave basis set with the kinetic cutoff energy of 400 eV, and the core electrons were replaced by the projector augmented wave (PAW) pseudopotentials.[46,50] Exchange and correlation were treated within the Perdew-Burke-Ernzerhof (PBE) generalized gradient approximation (GGA).[51] The k-point sampling was generated by following the Monkhorst-Pack procedure with a 3×3×1 mesh.[52] All structures were calculated until the Hellman-Feynman forces on each ion were lower than 0.03 eV/Å.

The adsorption energy (ΔE_{ads}) was calculated by using Eq(1), in which E_{total} was the total energy of the whole system upon adsorption, E_{mol} was the energy of the gas-phase molecule, and E_{slab} was the energy of the clean slab.

$$\Delta E_{ads} = E_{total} - (E_{slab} + E_{mol}) \quad \text{Eq(1)}$$

The differences of charge density (Δρ) of CO_2 adsorption were calculated according to the Eq(2).

$$\Delta\rho = \rho_{total} - (\rho_{slab} + \rho_{mol}) \quad \text{Eq(2)}$$

where ρ_{total}, ρ_{slab} and ρ_{mol} represented the total charge density of CO_2 adsorbed on Al_2O_3 surface, the charge density of clean Al_2O_3 surface and CO_2, respectively.

To evaluate the stability of different facets, we calculated the surface energies (ΔE_{surf}), see Eq(3):

$$\Delta E_{surf} = (E_{slab} - E_{bulk})/2A \quad \text{Eq(3)}$$

where E_{slab} and E_{bulk} denoted the energy of Al_2O_3 surface and bulk, respectively. A was the surface area of clean Al_2O_3 surface.

Theoretical models

The crystal structures of γ-Al_2O_3 are complicated. Usually, there are two kinds of structure models used by previous works, namely spinel type and nonspinel type.[45,53–55] The defective spinel type gamma-alumina (γ-Al_2O_3) as bulky model was chosen in this work.[56–59] Similar to previous theoretical works, the "Al_3O_4" bulk model was built by replacing all the Mg atoms in the $MgAl_2O_4$ (space group: *FD*-3M) by Al atoms.[60–62] The optimized lattice constant of resulting "Al_3O_4" was a=b=c=8.15 Å, which is in good agreement with previous works. The stoichiometric Al_2O_3(110) and Al_2O_3(100) surfaces were modeled by (2×1) and (1×2) supercells, respectively. The number of Al and O atoms on both surfaces are 48 and 72. To avoid the artificial interaction between the repeated slabs along z-direction, 15 Å of vacuum region is introduced. During the structural relaxation, all the atoms are fully optimized.

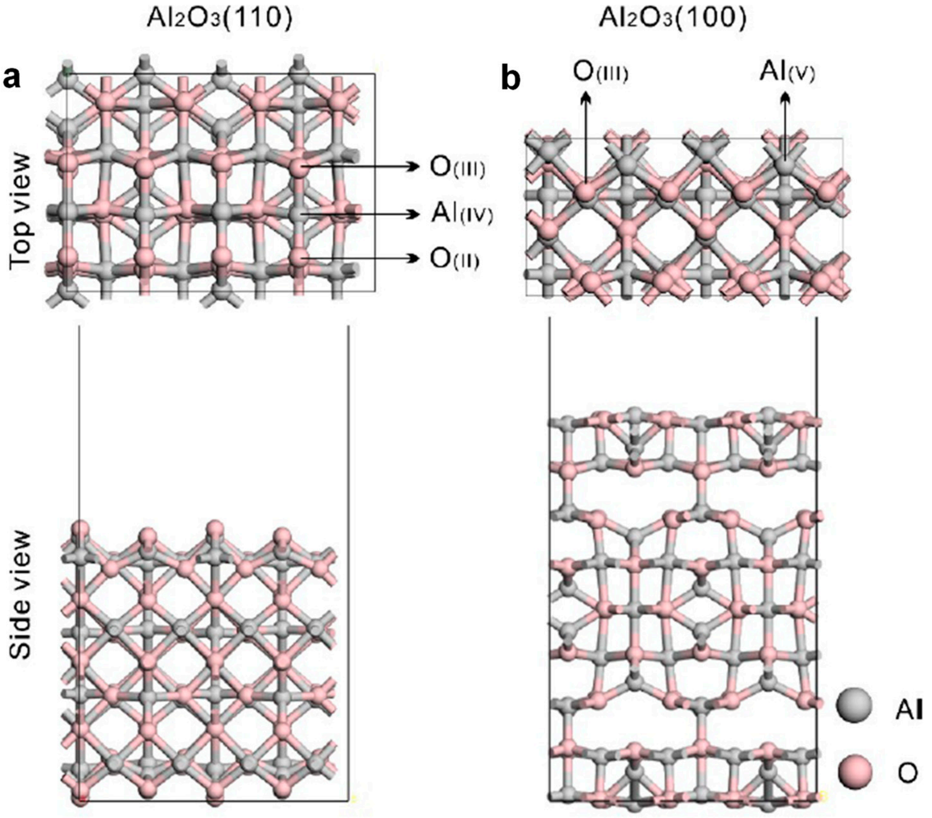

Figure S3. The top view and side view of the computational models. (a) Al_2O_3(110) and (b) Al_2O_3(100) surfaces.

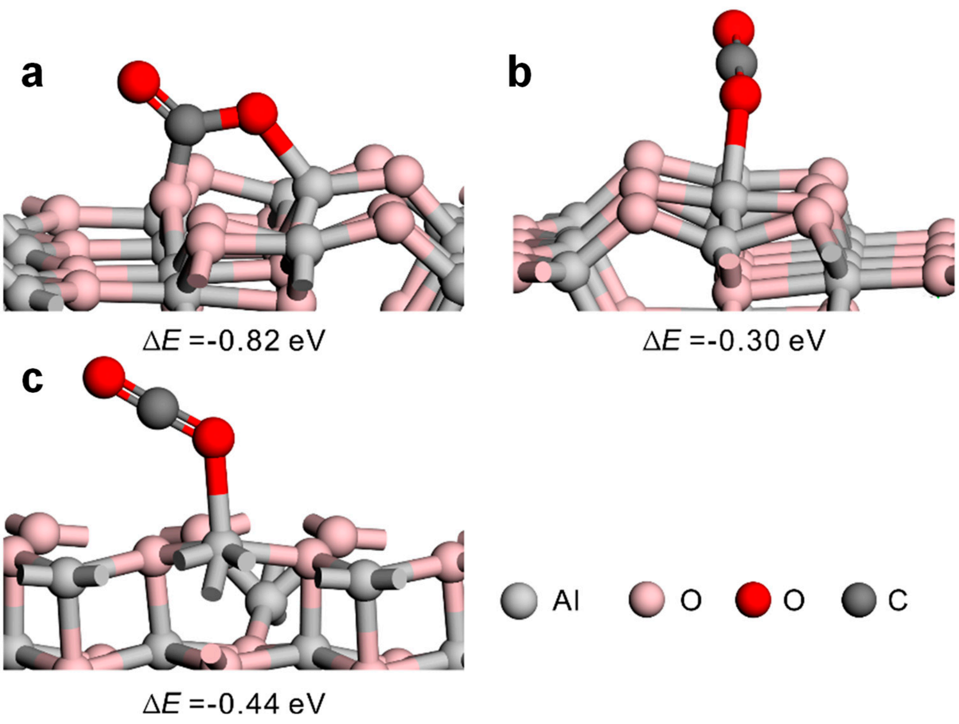

Figure S4. The adsorption energies and optimized structures of CO_2 on (a)-(b) Al_2O_3(110) and (c) Al_2O_3(100) surfaces.

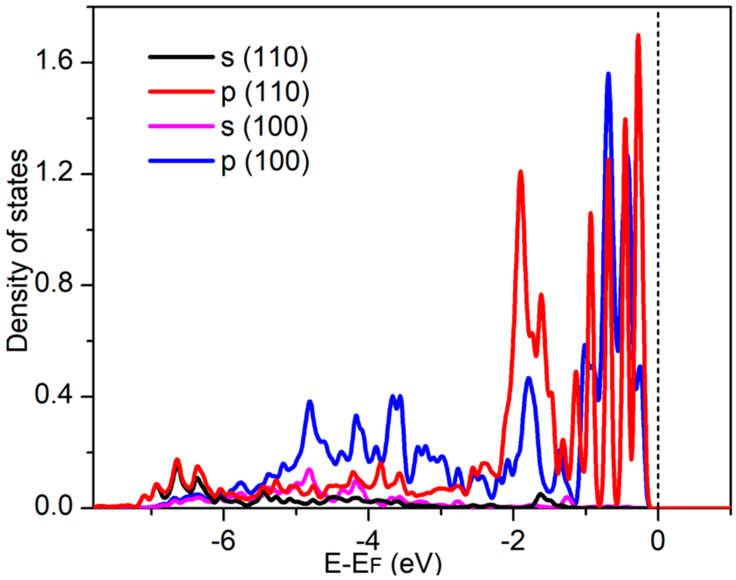

Figure S5. Projected density of states (PDOS) of the active sites of $Al_{(IV)}$-$O_{(II)}$ or $Al_{(V)}$-$O_{(III)}$ on Al_2O_3(110) and Al_2O_3(100), respectively.

The number of 2p electrons in the energy range from -2 eV to fermi level for active site atom of Al_2O_3(110) and Al_2O_3(100) is 1.03 a.u. and 0.84 a.u., respectively. The results indicate that the former surface is easier to transfer electrons to the antibonding molecular orbital of adsorbed CO_2.
